# Peptidorhamnomannans From *Scedosporium* and *Lomentospora* Species Display Microbicidal Activity Against Bacteria Commonly Present in Cystic Fibrosis Patients

**DOI:** 10.3389/fcimb.2020.598823

**Published:** 2020-10-28

**Authors:** Evely Bertulino de Oliveira, Mariana Ingrid Dutra da Silva Xisto, Rodrigo Rollin-Pinheiro, Victor Pereira Rochetti, Eliana Barreto-Bergter

**Affiliations:** Laboratório de Química Biológica de Microrganismos, Instituto de Microbiologia Paulo de Góes, Departamento de Microbiologia Geral, Universidade Federal do Rio de Janeiro (UFRJ), Rio de Janeiro, Brazil

**Keywords:** ****cystic fibrosis, peptidorhamnomannan, *Scedosporium*, bacteria, interaction

## Abstract

*Scedosporium* and *Lomentospora* species are filamentous fungi that cause a wide range of infections in humans. They are usually found in the lungs of cystic fibrosis (CF) patients and are the second most frequent fungal genus after *Aspergillus* species. Several studies have been recently performed in order to understand how fungi and bacteria interact in CF lungs, since both can be isolated simultaneously from patients. In this context, many bacterial molecules were shown to inhibit fungal growth, but little is known about how fungi could interfere in bacterial development in CF lungs. *Scedosporium* and *Lomentospora* species present peptidorhamnomannans (PRMs) in their cell wall that play crucial roles in fungal adhesion and interaction with host epithelial cells and the immune system. The present study aimed to analyze whether PRMs extracted from *Lomentospora prolificans*, *Scedosporium apiospermum*, *Scedosporium boydii*, and *Scedosporium aurantiacum* block bacterial growth and biofilm formation *in vitro*. PRM from *L. prolificans* and *S. boydii* displayed the best bactericidal effect against methicillin resistant *Staphylococcus aureus* (MRSA), *Burkholderia cepacia*, and *Escherichia coli*, but not *Pseudomonas aeruginosa*, all of which are the most frequently found bacteria in CF lungs. In addition, biofilm formation was inhibited in all bacteria tested using PRMs at minimal inhibitory concentration (MIC). These results suggest that PRMs from the *Scedosporium* and *Lomentospora* surface seem to play an important role in *Scedosporium* colonization in CF patients, helping to clarify how these pathogens interact to each other in CF lungs.

## Introduction

Cystic Fibrosis (CF) is an autosomal recessive disease originated from a mutation in the gene encoding the cystic fibrosis transmembrane conductance regulator (CFTR), a cAMP-regulated epithelial chloride channel (Cohen and Prince, 2012). It results in deficient mucociliary functions, leading to the presence of thicker mucus especially in bronchia and pancreas and, consequently, patients suffer of digestive and respiratory problems (Delhaes et al., 2012). The occurrence of CF is estimated to be one in 3,000 or 4,000 births, and one in 25–30 Caucasians carries the mutation in CFTR gene. In the USA about 1,000 people are diagnosed every year, with CF (Sanders and Fink, 2016).

Sticky bronchial mucus is the most challenging problem in CF patients, because it facilitates the occurrence of airway infections and neutrophilic inflammation that are mostly responsible for morbidity and death in these patients (Stoltz et al., 2015). For these reasons, chronic pulmonary infections are frequent and extensively studied. Several pathogens including bacteria and fungi are commonly associated with CF lung colonization. Bacterial pathogens are the most common cause of CF lung infections, in which *Staphylococcus aureus* and *Haemophilus influenza* are frequent in children and *Burkholderia cepacia* complex and *Pseudomonas aeruginosa* are found especially in adults (Ciofu et al., 2013; Vandeplassche et al., 2019). However, fungal colonization is usually found in the lungs of CF patients, although its dynamics are less clear (Middleton et al., 2013). In this context, the most frequent fungi are *Aspergillus*, *Penicillium*, *Scedosporium*, and *Candida* species (Ziesing et al., 2016; Engel et al., 2019).

For these reasons, polymicrobial colonization is a well-known situation found in CF lungs and the interaction between bacteria and fungi is a field of extensive studies in order to better understand the complexity and dynamics of polymicrobial relationship in this context (Delhaes et al., 2012). A variety of studies demonstrate that bacteria produce molecules known to inhibit fungal growth. Phenazine and other secreted molecules, for instance, are produced by *P. aeruginosa* and inhibit *Aspergillus fumigatus*, *Candida albicans*, and *Scedosporium* species (Gibson et al., 2009; Mowat et al., 2010; Kaur et al., 2015; Chen et al., 2018; Briard et al., 2019). On the other hand, little is known about how fungi can affect bacterial growth in CF conditions. *C. albicans* inhibits *P. aeruginosa* growth by producing farnesol, a quorum-sensing molecule which plays a role in fungal morphogenesis (Semighini et al., 2006).

In the context of fungal molecules that could influence bacterial growth, the present work studied peptidorhamnomannans (PRMs), glycoconjugates commonly exposed on *Scedosporium* and *Lomentospora* cell wall that play important roles in cell adhesion and interaction with host immune system, leading to the release of inflammatory cytokines by phagocytic cells (Lopes et al., 2011). PRMs have been already described and characterized in *Scedosporium boydii*, *Scedosporium apiospermum*, and *Lomentospora prolificans* (Pinto et al., 2001; Lopes et al., 2010; Xisto et al., 2015), as well as in *Scedosporium aurantiacum* (de Meirelles et al., 2020). The PRMs of these four species possess similar epitopes as well as distinct species-specific oligosaccharide chains. For these reasons, in the present work PRMs extracted and purified from these four species were tested for their effect on bacterial species relevant to CF, such as *P. aeruginosa*, *B. cepacia*, methicillin-resistant *S. aureus*, and *Escherichia coli*.

## Material and Methods

### Microorganisms and Culture Conditions

*B. cepacia* (American Type Culture Collection ATCC 25416), *E. coli* (ATCC 11229), Methicillin Resistant *S. aureus*—MRSA (ATCC 9393) and *P. aeruginosa* (ATCC 27853) were studied in this work. Strains were maintained in Luria-Bertani (LB) broth medium (peptone 10 g/l, yeast extract 5 g/l and NaCl 5 g/l) under refrigeration at 4°C. For recent overnight cultures, an aliquot of each strain was spread on LB agar and incubated for 24 h at 37°C, and bacterial suspensions were prepared from scrapings.

*L. prolificans* (FMR3569 strain), *S. apiospermum* (RK107-0417 strain) and *S. aurantiacum* (IHEM21147 strain) were supplied by Dr. J. Guarro, Unitat de Microbiologia, Facultat de Medicina e Institut d`Estudis Avançats, Réus, Spain. *S. boydii* (HLPB strain) was supplied by Dr. Bodo Wanke, Hospital Evandro Chagas, Instituto Oswaldo Cruz, Rio de Janeiro, Brazil. Strains were maintained at room temperature on Sabouraud (SAB; 2% glucose, 1% peptone, 0.5% yeast extract) agar slants as stock culture. Mycelia were obtained by growing cells in SAB liquid culture medium for seven days at room temperature with shaking.

### Extraction and Purification of Peptidorhamnomannan (PRM)

The crude glycoprotein was extracted from *L. prolificans*, *S. apiospermum*, *S. boydii*, and *S. aurantiacum* with 0.05M phosphate buffer, pH 7.2, at 100°C for 2 h. After filtration, the solution was dialyzed, evaporated to a small volume, and lyophilized. Peptidorhamnomannans were purified by hexadecyltrimethylammonium bromide (Cetavlon, Merck, Darmstadt, Germany) fractionation, according to Barreto-Bergter et al. (2008).

### Determination of MIC and MBC

Minimum inhibitory concentration (MIC) of the PRM isolated from *L. prolificans*, *S. apiospermum*, *S. boydii*, and *S. aurantiacum* was determined through broth microdilution method in LB broth against bacteria, according to Vieira et al. (2018). MIC was defined as the lowest concentration capable of inhibiting 50% of bacterial growth (MIC%). Tests were performed on 96-well microplates. PRM at final concentrations ranging from 500 μg/ml to 1.95 μg/ml in LB broth medium were added to each well (50 µl/well). From recent overnight cultures, 50 µl of bacterial suspensions with turbidity equivalent to 0.5 tube of the McFarland scale (1.5 x 10^8^ CFU/ml) were added in each well and incubated at 37°C for 24 h. Streptomycin/penicillin was used as reference compounds (8–0.015 µg/ml) and cultures in LB broth without antibiotics were used as control. After determining MIC values, MBC (minimum bactericidal concentration) was determined by subculturing an aliquot of 10 µl from each well that showed complete growth inhibition in LB agar medium without addition of PRM and evaluation of bacterial growth (Vieira et al., 2018). After 24 h, MBC values were defined as the lowest concentration of PRM able to kill bacteria.

### Biofilm Assay

#### Inhibition of Biofilm Formation

For biofilm formation, 50 µl of *B. cepacia* (ATCC 25416) and MRSA (ATCC9393) bacterial solutions (prepared as described in MIC section) were added in a 96-well plate and mixed with 1/4, 1/2 and 1 MIC of each PRM. After 24 h of incubation at 37°C, the formed biofilm was gently washed with PBS to remove planktonic cells, air-dried for 10 min and stained for 10 min with 0.5% crystal violet (total biofilm biomass) or 1% safranin (biofilm matrix). The staining solutions were discarded and biofilms were gently rinsed twice with sterile distilled water. Crystal violet impregnated in the biofilm was dissolved in 200 µl of ethanol (95%, v/v), and the colored solution was read at an absorbance of 595 nm using a spectrophotometer (Spectra MAX 340 Tunable; Molecular Devices Ltd., San Jose, CA, USA). Safranin was dissolved in water (100 µl), and the absorbance read at 492 nm.

#### Inhibition of Preformed Biofilm

The *B. cepacia* and MRSA biofilms were prepared as described in the previous section, and after incubation for 24 h at 37°C, the supernatant was removed and 1/2, 1 and 2 MICs of each PRM were added to the formed biofilm. After 24 h at 37°C, the biofilm was gently washed with PBS and crystal violet and safranin staining were performed as in the previous section.

### Effect of PRM on ROS Production and Membrane Potential and Integrity

In order to evaluate the effect of fungal PRMs on oxidative stress and membrane integrity and potential, *B. cepacia* cells were grown in LB broth in the presence of ¼ MIC of all four PRMs for 24 h at 37°C. Furthermore, fluorescence staining was performed using DCFDA (2’,7’-dichlorodihydrofluorescein diacetate), Nile Red and JC-1 (5,5’,6,6’-tetrachloro-1,1’,3,3’tetraethylbenzimidazolylcarbocyanine iodide) (both from Sigma-Aldrich, MO, USA), according to Ullrich et al. (2003); Pereira et al. (2007) and Van Acker et al. (2016), with modifications.

Staining with DCFDA was performed at a final concentration of 10 μM in PBS for 45 min at room temperature in the dark. After washing with PBS, fluorescence (λ excitation = 485 nm, λ emission = 535 nm) was measured using a SpectraMax plate reader (Van Acker et al., 2016).

Nile Red staining at a final concentration of 8 μg/ml was done for 45 min at room temperature in the dark. After washing with PBS, fluorescence (λ excitation = 550 nm, λ emission = 635 nm) was measured as above (Pereira et al., 2007).

JC-1 stain was used at 2.5 μg/ml in PBS 0.01 M, pH 7.2, for 45 min at 37°C in the dark. After washing with PBS, fluorescence (λ excitation = 515 nm, λ emission = 529 nm (green) and 590 nm (red) was measured as above (Ullrich et al., 2003). The membrane potential was determined by the red/green fluorescence intensity ratio.

### Statistical Analysis

All statistical analyses were performed using GraphPad Prism 5.0 software (GraphPad, San Diego, CA, USA). A variance two-way ANOVA was performed using Tukey’s and Bonferroni’s comparisons tests to evaluate biofilm formation and inhibition of preformed biofilm.

## Results

### Minimum Inhibitory and Bactericidal Concentrations (MIC and MBC) of PRMs

MIC and MBC values were determined for PRMs isolated from *L. prolificans*, *S. apiospermum*, *S. boydii*, and *S. aurantiacum* against *B. cepacia*, *E. coli*, MRSA, and *P. aeruginosa* (**Table 1**). *L. prolificans* PRM showed considerable inhibitory activity against *B. cepacia* and MRSA, with MIC of 31.3 and 15.6 µg/ml, respectively. However, for *E. coli* MIC was 500 µg/ml. *S. apiospermum* PRM present MIC of 500 µg/ml for *B. cepacia* and MRSA, and >500 µg/ml for *E. coli*. *S. boydii* PRM displayed MIC of 125 µg/ml for *B. cepacia*, 125 µg/ml for MRSA and >500 µg/ml for *E. coli*. *S. aurantiacum* PRM showed MIC at 125 µg/ml for *B. cepacia* and MRSA, and >500 µg/ml for *E. coli*. All PRMs tested displayed MIC at >500 µg/ml for *P. aeruginosa*.

**Table 1:** Minimal inhibitory concentration (MIC) and minimal bactericidal concentration (MBC) of fungal PRMs isolated from *L. prolificans*, *S. apiospermum*, *S. boydii* and *S. aurantiacum* tested against *B. cepacia*, *E. coli*, MRSA and *P. aeruginosa*.

Bacteria	PRM (µg/ml)							
	*L. prolificans*		*S. apiospermum*		*S. boydii*		*S. aurantiacum*	
	MIC_50_	MBC	MIC_50_	MBC	MIC_50_	MBC	MIC_50_	MBC
*B. cepacia*	31.3	31.3	500	>500	125	500	125	> 500
*E. coli*	500	> 500	> 500	> 500	> 500	> 500	> 500	> 500
MRSA	15.6	31.3	500	>500	125	250	125	> 500
*P. aeruginosa*	>500	>500	>500	>500	>500	>500	> 500	> 500

MIC50, minimal concentration that inhibits 50% of growth; MBC, minimal bactericidal concentration; MRSA, methicillin resistant S. aureus.

Regarding MBC (**Table 1**), *L. prolificans* PRM was found to be bactericidal at 31.3 µg/ml for *B. cepacia* and MRSA, at 500 µg/ml for *E. coli*, but was not able to kill *P. aeruginosa*. *S. boydii* PRM displayed MBC at 500 µg/ml for *B. cepacia* and 250 µg/ml for MRSA, and was not able to kill either *E. coli* or *P. aeruginosa*. *S. apiospermum* and *S. aurantiacum* PRMs did not show bactericidal activity for any bacterium tested. Supplementary Material shows bacteria incubated with different concentrations of all PRMs. After 24h at 37°C, aliquots were spotted on LB agar in the absence of any PRM, in order to determine MBCs shown in **Table 1**.

These results indicate that *L. prolificans* PRM was most active against bacteria compared to the other PRMs, whereas *S. apiospermum* PRM was the less potent molecule to inhibit bacterial proliferation. In addition, *B. cepacia* and MRSA were the most susceptible species when incubated with all four PRMs.

### Inhibition of Biofilm Formation

**Figure 1:**
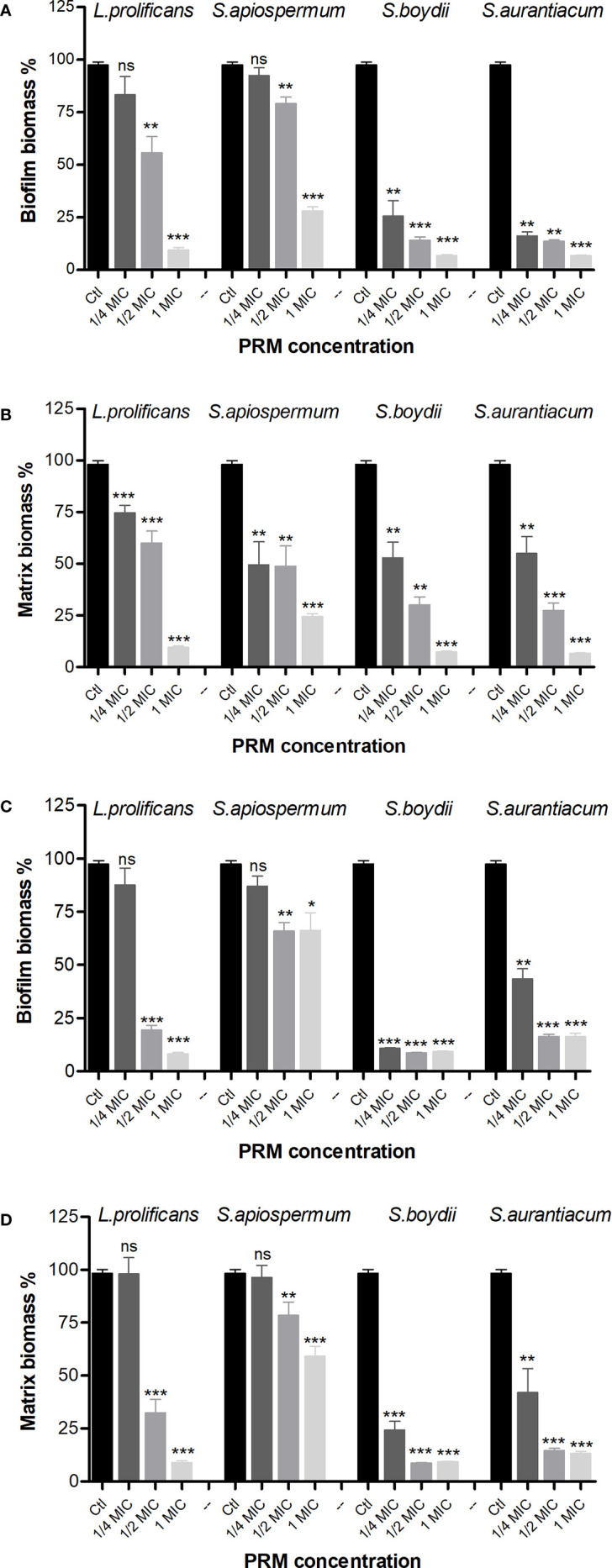
Biofilm formation of *B cepacia* **(A, B)** and MRSA **(C, D)** in the presence of PRMs isolated from *L. prolificans*, *S. apiospermum*, *S. boydii* and *S. aurantiacum*. **(A, C)** represents the evaluation of biofilm growth. **(B, D)** show extracellular matrix. Ctl, control without addition of PRM. MIC, ¼ MIC and ½ MIC were based on the values shown in **Table 1**. The values represent the mean ± S.D. of three independent experiments performed in triplicate. Asterisks denote values statistically different from control. *p < 0.05; **p < 0.01; ***p < 0.001. ns, no significant.

Since *B. cepacia* and MRSA were the species most susceptible to fungal PRMs, we chose these two species to evaluate whether fungal PRMs could also affect bacterial biofilm formation. In this context, the total formed biomass and the extracellular matrix were evaluated. When *B. cepacia* was analyzed, *L. prolificans* and *S. apiospermum* PRMs could inhibit biomass formation at MIC and ½ MIC (**Figure 1A**). However, PRM isolated from *S. boydii* and *S. aurantiacum* showed a significant inhibition of *B. cepacia* biomass formation at MIC, ½ MIC and also ¼ MIC (**Figure 1A**). Biofilm matrix was reduced by all PRMs at 1 MIC, ½ MIC and ¼ MIC (**Figure 1B**).

Regarding biofilm formation by MRSA, similar results were observed for biomass (**Figure 1C**). However, extracellular matrix was reduced at MIC and ½ MIC by *L. prolificans* and *S. apiospermum* PRMs, and at all three concentrations used for *S. boydii* and *S. aurantiacum* PRMs (**Figure 1D**).

These data suggest that besides growth inhibition observed in MIC and MBC experiments, biofilm formation is also decreased when bacteria are grown in the presence of fungal PRM.

### Inhibition of Preformed Bacterial Biofilm

**Figure 2:**
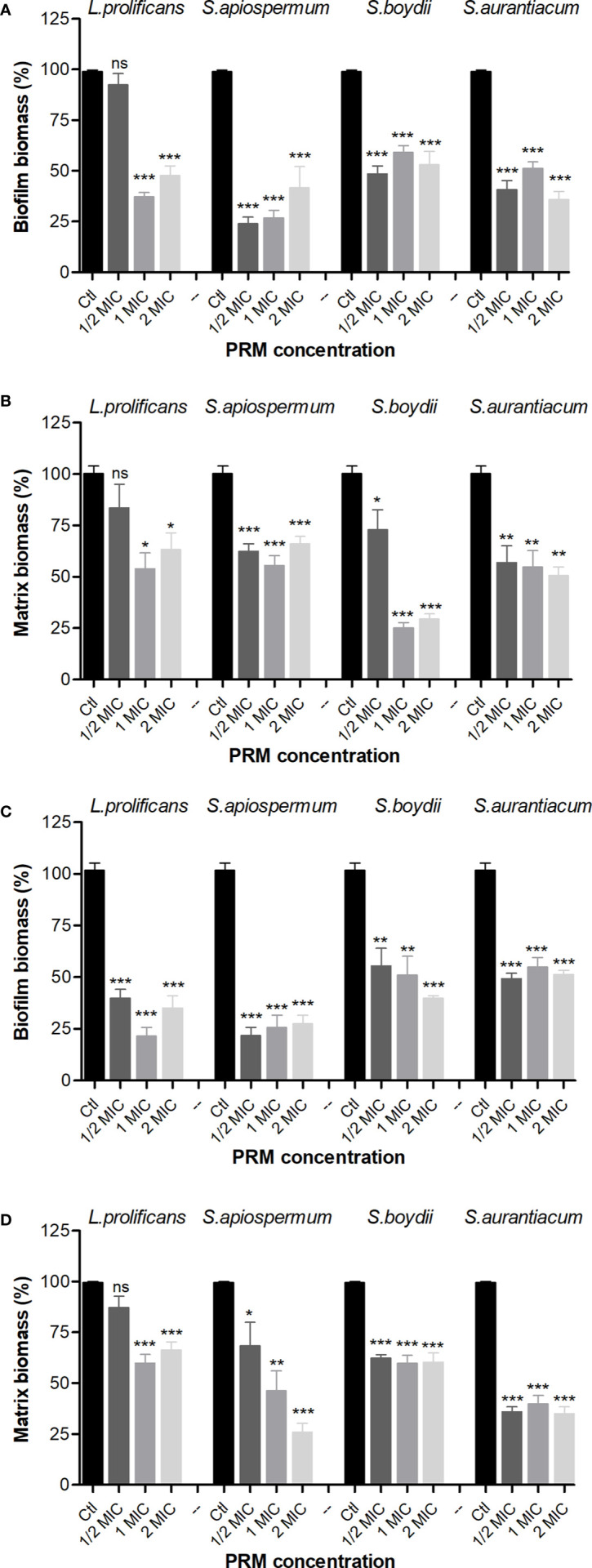
Effect of PRMs isolated from *L. prolificans*, *S. apiospermum*, *S. boydii* and *S. aurantiacum* on preformed biofilm of *B cepacia* **(A, B)** and MRSA **(C, D)**. **(A, C)** represent the evaluation of biofilm formation. **(B, D)** show extracellular matrix. Ctl, control without addition of PRM. MIC, ¼ MIC and ½ MIC were based on the values shown in **Table 1**. The values represent the mean ± S.D. of three independent experiments performed in triplicate. Asterisks denote values statistically different from control. *p < 0.05; **p < 0.01; ***p < 0.001. ns, no significant.

Due to the inhibitory potential of fungal PRMs on bacterial growth and biofilm formation, we decided to check whether these four PRMs could also be active against preformed biofilms. *L. prolificans* PRM reduced preformed biomass and extracellular matrix of *B. cepacia* at MIC and ½ MIC, whereas PRMs isolated from *S. apiospermum*, *S. boydii* and *S. aurantiacum* showed similar results at all concentrations tested (**Figures 2A, B** **)**. When preformed MRSA biofilm was evaluated, all four PRMs decreased biomass matrix at all concentrations used (**Figures 2C, D** **)**, except for *L. prolificans* PRM at ¼ MIC, which was not able to significantly reduce MRSA matrix (**Figure 2D**).

These results suggest that fungal PRMs affect not only bacterial growth and the process of biofilm formation, but they affect also preformed bacterial biofilms.

### PRMs Effect on ROS Production, Membrane Potential, and Integrity

In order to understand the effects of PRMs on bacteria, oxidative stress was evaluated in bacterial cells, as well as the integrity and the potential of the membrane. To perform these analyses, *B. cepacia* was chosen as a representative model due to its relevance in CF patients with worse prognosis.

**Figure 3:**
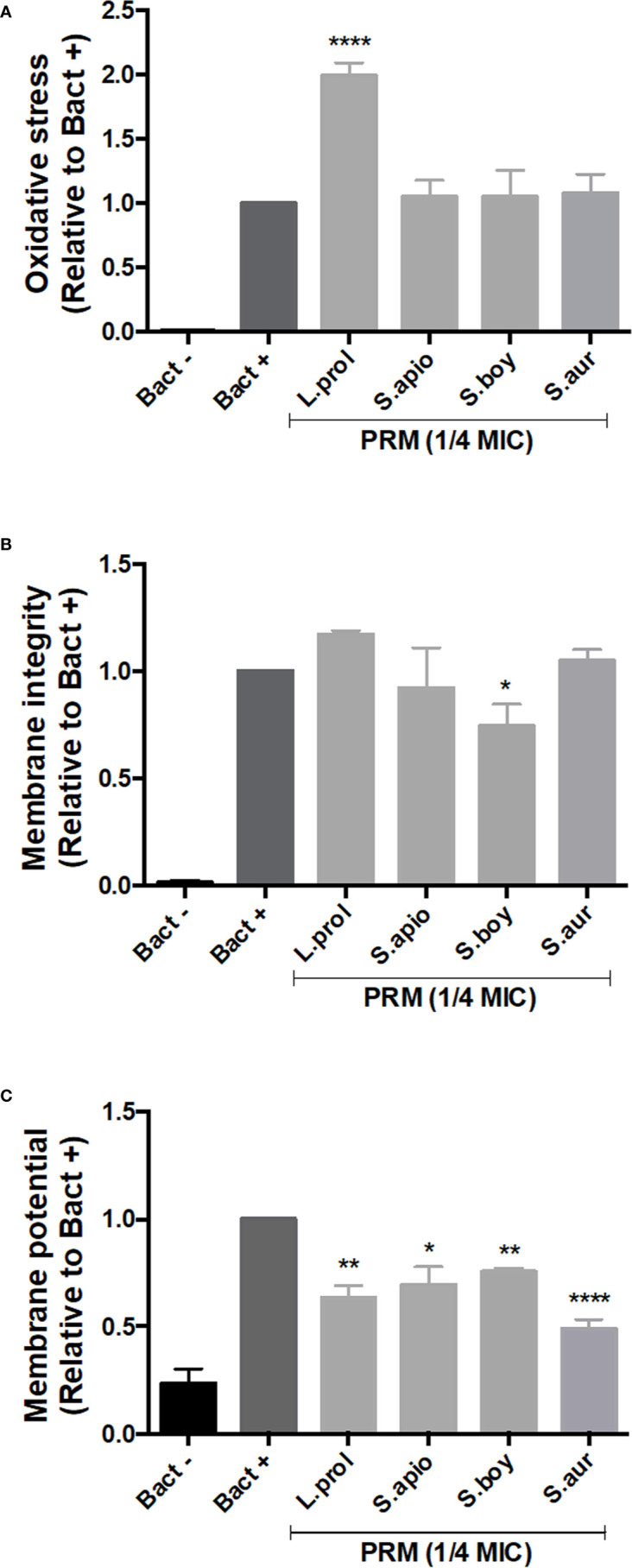
Effect of PRMs isolated from *L. prolificans*, *S. apiospermum*, *S. boydii* and *S. aurantiacum* on oxidative stress **(A)**, membrane integrity **(B)** and membrane potential **(C)** of *B cepacia*. Oxidative stress, membrane integrity and potential were measured by DCFDA, Nile Red and JC-1 staining, respectively. Bact-, unstained control. Bact+, stained cells in the absence of PRM. L. prol, *L prolificans*. S. apio, *S. apiospermum*. S. boy, *S. boydii*. S. aur, *S. aurantiacum*. ¼ MIC was based on the values shown in **Table 1**. The values represent the mean ± S.D. of three independent experiments performed in triplicate. Asterisks denote values statistically different from control. *p < 0.05; **p < 0.01; ****p < 0.001.

The production of reactive oxygen species (ROS), which provoke oxidative stress, was measured using DCFDA staining. DCFDA is oxidized by ROS inside the cells to form fluorescent 2’, 7’-dichlorofluorescein (DCF). In this context, *L. prolificans* PRM was the only molecule able to increase ROS production, indicating induction of oxidative stress in *B. cepacia* (**Figure 3A**). The other PRMs did not influence ROS production when compared to the control.

Disruption of membrane integrity is a mechanism by which some compounds cause cell killing. For this reason, we used Nile Red staining that binds to polyhydroxyalkanoates (PHAs), common prokaryotic storage compounds of carbon and energy present in intracellular lipid droplets (Jendrossek, 2009). *S. boydii* PRM decreased Nile Red fluorescent intensity, suggesting that it affects lipid integrity in *B. cepacia*, whereas the other PRMs did not alter bacterial lipids when compared to the control (**Figure 3B**).

Reduction of energy generation as consequence of cell killing could be measured by evaluating the membrane potential using JC-1 staining. All fungal PRMs tested caused a decrease in membrane potential compared to the control (untreated cells), indicating that PRMs induce a lower membrane polarization in *B. cepacia* (**Figure 3C**).

## Discussion

Cystic fibrosis patients exhibit a reduced clearance of mucus from the lungs which leads to chronic infections caused by bacteria and fungus, being the primary cause of mortality (Lipuma, 2010; Cohen and Prince, 2012). The study of polymicrobial infections in this environment is not only important for understanding the pathogenesis but also for providing insights for the development of new antimicrobial drugs, since the relationships between these microorganisms in the context of CF are frequently antagonistic and therefore may reveal new approaches for pathogen inhibition (Peleg et al., 2010).

Bacteria play a major part in lungs of CF patients. They are usually the first microorganisms to colonize this site and are the cause of a common exacerbated inflammatory response observed in the respiratory tract. Guss et al. (2011) identified eight bacterial phyla, including more than 60 genera, in lungs from CF patient, therefore evidencing the diversity of this microbial community. However, as previously mentioned, there are some more prevalent species colonizing the pulmonary tract in CF, such as *P. aeruginosa*, *S. aureus*, and *B. cepacia*. The presence of *B. cepacia* is considered a threat to CF patients, due to its high patient-to-patient transmissibility and antibiotic resistance, as well as a poorer prognosis of the disease outcome (Kenna et al., 2017). A recent study showed that *E. coli* was recovered from the sputum of up to 25% of patients with CF, mainly from those with poor nutritional status and lung function, although this did not predict clinical decline (Edwards et al., 2020). In our study, *P. aeruginosa*, *S. aureus*, *B. cepacia*, and *E. coli* were used as representative species for CF to evaluate the bacterial inhibitory effect of fungal PRMs isolated from *L. prolificans*, *S. boydii*, *S. apiospermum*, and *S. aurantiacum*.

Bacteria are the main cause of CF lung infections, but fungal species are also able to chronically colonize the respiratory tract of these patients. It is known that fungi from the genus *Scedosporium* are the third most prevalent in CF patients after *Aspergillus* and *Penicillium*. *S. apiospermum* is the most frequent (28.6%), followed by *S. boydii* (19.3%), *S. aurantiacum* (10.0%), and *L. prolificans* (3.6%) (Engel et al., 2019). Other studies described *Scedosporium* species as the second most prevalent filamentous fungi in CF patients (Pihet et al., 2009; Sudfeld et al., 2010).

Considering the polymicrobial pattern of CF lung infections, it is relevant to study the bacteria-fungi interactions and the molecules produced by fungi that could be involved in these interactions. In this context, the present work used PRMs isolated from *L. prolificans* (Barreto-Bergter et al., 2008), *S. boydii* (Pinto et al., 2005), *S. apiospermum* (Barreto-Bergter et al., 2011), and *S. aurantiacum* (de Meirelles et al., 2020). These glycoconjugates are exposed on fungal surfaces, since they are recognized by antibodies anti-PRM (Lopes et al., 2010; Xisto et al., 2015). Cell culture supernatants of *Scedosporium* and *Lomentospora* species for the presence of PRM were not examined in this manuscript. *In vitro* studies show that variable amounts of galactomannan (GM), a component of the cell wall of several *Aspergillus* species as well of a diverse range of fungi, are released during early logarithmic growth (Latgé et al., 1994). *In vivo* it is known that galactomannans can be detected in the bronchoalveolar lavage of patients with invasive aspergillosis, released from *A. fumigatus* cell wall during infections (Sarfati et al., 1995; He et al., 2012; Teering et al., 2014). Further studies, not only focused on the characterization of extracellular PRM from *Scedosporium* and *Lomentospora* species but also on carbohydrate-containing molecules isolated from cell wall or secreted into the cell culture supernatant of these species cultivated in synthetic cystic fibrosis sputum medium (SCFM) (Han et al., 2017), that mimics the environment in the CF lungs species will improve the knowledge of this complex bacteria-fungus interaction in CF.

In addition, PRMs of *S. boydii* and *S. apiospermum* are involved in the adhesion to an epithelial cell line and in intracellular survival within macrophages, respectively (Pinto et al., 2004; Lopes et al., 2010). PRMs isolated from *S. boydii* and *L. prolificans* also play a role in host immune stimulation, increasing the release of pro-inflammatory cytokines such as TNF-α (Figueiredo et al., 2010; Xisto et al., 2015).

Taken together, all data presented in our study demonstrated that PRMs possess antibacterial and anti-biofilm effects. Regarding the inhibition of bacterial growth, PRM from *L. prolificans* was the most effective, followed by those from *S. aurantiacum*, *S. boydii*, and *S. apiospermum*, against *B. cepacia* and MRSA, which were the most susceptible bacteria. *P. aeruginosa* and *E. coli* seemed to be resistant to all PRM. Considering the anti-biofilm effect, PRM from *S. boydii* and *S. aurantiacum* showed to be the most effective, because ¼ MIC was necessary to observe *B. cepacia* and MRSA biofilm reduction.

Although *S. apiospermum* is described as the most common *Scedosporium* species identified in lungs of CF patients, MIC and MBC results obtained in this work showed that its PRM was the less potent molecule against bacteria, whereas *L. prolificans* PRM, which is the less frequent fungi of *Scedosporium/Lomentospora* group isolated from CF patients, was the most active against bacteria. In addition, all PRMs presented high MICs against *P. aeruginosa* and *E. coli*. Rhamnose-containing molecules, such as the rhamnolipid biosurfactant isolated from a sponge associated marine fungus *Aspergillus* spp has been described with antimicrobial activity against *C. albicans* and some Gram-negative bacteria (Kiran et al., 2009).

Variation in the antibacterial potential among PRMs could be due to differences in their oligosaccharide chains. PRMs isolated from the *Scedosporium* and *Lomentospora* species used in this work share similar epitopes, such as the α-Rhap-(1→3) α-Manp-(1→2)- α-Manp (Lopes et al., 2011; de Meirelles et al., 2020). On the other hand, structural differences are observed among these PRMs, which could result in changes in the antimicrobial activity. *L. prolificans* PRM, for instance, contains a pentasaccharide lacking the β-Galp side-chain (Barreto-Bergter et al., 2008) and a high proportion of 2-*O*-substituted Rhap units, which are absent in *S. boydii* and *S. apiospermum* PRMs (Pinto et al., 2005; Barreto-Bergter et al., 2008; Barreto-Bergter et al., 2011). Nevertheless, more studies are needed in order to clarify the reason why *L. prolificans* PRM present a higher activity against the bacteria tested when compared to the other PRMs.

Due to the relevance of biofilms in infection, the influence of PRM on bacterial biofilms was also evaluated. All four PRMs inhibit biofilm formation and are active against preformed biofilms of *B. cepacia* and MRSA at MIC values. Extracellular matrix was also reduced by PRMs, indicating that bacterial biofilms were weakened in the presence of PRM. Several glycoconjugates and polysaccharides have been already described as potent inhibitors of bacterial biofilms. C-fucosylpeptide and galactosylated peptide dendrimers have been shown to inhibit biofilm formation and to disperse preformed biofilm by interfering with *P. aeruginosa* LecA and B, a lectin molecule responsible for bacterial adherence on host tissues (Kadam et al., 2011; Reymond et al., 2013). Human and plant oligosaccharides, such as galactooligosaccharides, have already been known to block bacterial adhesion to surfaces, decreasing biofilm formation of *E. coli*, *Burkholderia pseudomallei* and other bacteria (Thomas and Brooks, 2004; Shoaf et al., 2006; Lane et al., 2010; Quintero et al., 2011; Rendueles et al., 2013). Regarding fungal molecules, chitosan, a deacetylated derivative of chitin found on the fungal cell wall is recognized as a potent antibacterial agent and inhibits growth and biofilm formation by different bacteria, such as *S. aureus* and *E. coli* (Asli et al., 2017). For other fungal glycoconjugates, such as the rhamnolipid biosurfactant of *Aspergillus* spp., antimicrobial activity against *Streptococcus* spp., *Micrococcus luteus*, and *Enterococcus faecalis* has also been reported (Kiran et al., 2009).

In order to evaluate some possible mechanisms involved in bacterial death, we evaluated oxidative stress, membrane integrity and membrane potential in *B. cepacia* treated with PRMs using DCFDA, Nile Red and JC-1 staining, respectively. *L. prolificans* PRM increased ROS production when compared to control. PRMs isolated from *S. boydii*, *S. apiospermum* and *S. aurantiacum* did not cause any effect, suggesting that structural differences among PRMs might be related to ROS production by *B. cepacia*. On the other hand, a decrease of the membrane potential was observed in *B. cepacia* in the presence of all PRMs used. Membrane integrity was only affected when bacterial cells were treated with *S. boydii* PRM. Membrane alteration detected by Nile Red is well known in bacteria treated with penicillin, which leads to disorganized cell surface (Pereira et al., 2007). However, few information about ROS induction, membrane disorganization and changes in membrane potential caused by glycoconjugates and polysaccharides is described in the literature.

The knowledge of how fungi can influence bacterial growth in the polymicrobial colonization of lungs from CF patients is still scarce. Several studies have been performed in order to understand how bacteria, especially *P. aeruginosa*, inhibit competitors in CF airways (Gibson et al., 2009; Mowat et al., 2010; Delhaes et al., 2012; Kaur et al., 2015; Chen et al., 2018; Briard et al., 2019). However, studies on the mechanisms by which fungal molecules inhibit bacteria are rare. Therefore, the present work aimed to evaluate the effect of the main glycoconjugate found on the *Scedosporium* and *Lomentospora* cell wall on bacterial growth. PRMs from *Scedosporium* and *Lomentospora* species were able to kill bacteria associated to lungs of CF patients and interfere with both, bacterial biofilm formation and preformed biofilms. The mechanisms involved in *B. cepacia* death are different among the PRMs tested in this work, and can be associated with structural differences in these PRMs. Since CF prognosis is hard for all patients and infections of the respiratory tract are the main cause of mortality in this population, studies that contribute to the understanding of the dynamics of bacteria-fungi interactions in the context of CF are crucial for developing better approaches to increase the patients’ quality of life.

## Data Availability Statement

The raw data supporting the conclusions of this article will be made available by the authors, without undue reservation.

## Author Contributions

EO, MX, RRP, and VR conceived, designed, and performed the experiments. EO, MX, RRP, VR, and EBB analyzed the experiments. MX, RRP, and EBB drafted the manuscript. All authors contributed to the article and approved the submitted version.

## Funding

This study was financed in part by the Coordenação de Aperfeiçoamento de Pessoal de Nível Superior—Brasil (CAPES)—Finance Code 001; Conselho Nacional de Desenvolvimento Científico e Tecnológico (CNPq) and Fundação de Amparo à Pesquisa do estado do Rio de Janeiro (Faperj).

## Conflict of Interest

The authors declare that the research was conducted in the absence of any commercial or financial relationships that could be construed as a potential conflict of interest.

## Supplementary Material

Supplementary Material 1 Growth of *B. cepacia*, *E. coli*, MRSA and *P. aeruginosa* in the presence of different concentrations (0.98 – 500 μg/ml) of PRM isolated from *L. prolificans*, *S. apiospermum*, *S. boydii* and *S. aurantiacum*.
