# Supplementary figures and images for: Peptidorhamnomannans From Scedosporium and Lomentospora Species Display Microbicidal Activity Against Bacteria Commonly Present in Cystic Fibrosis Patients
Source: Front Cell Infect Microbiol. 2020 Oct 28;10:598823. doi: 10.3389/fcimb.2020.598823 (PMC7673444; doi:10.3389/fcimb.2020.598823)

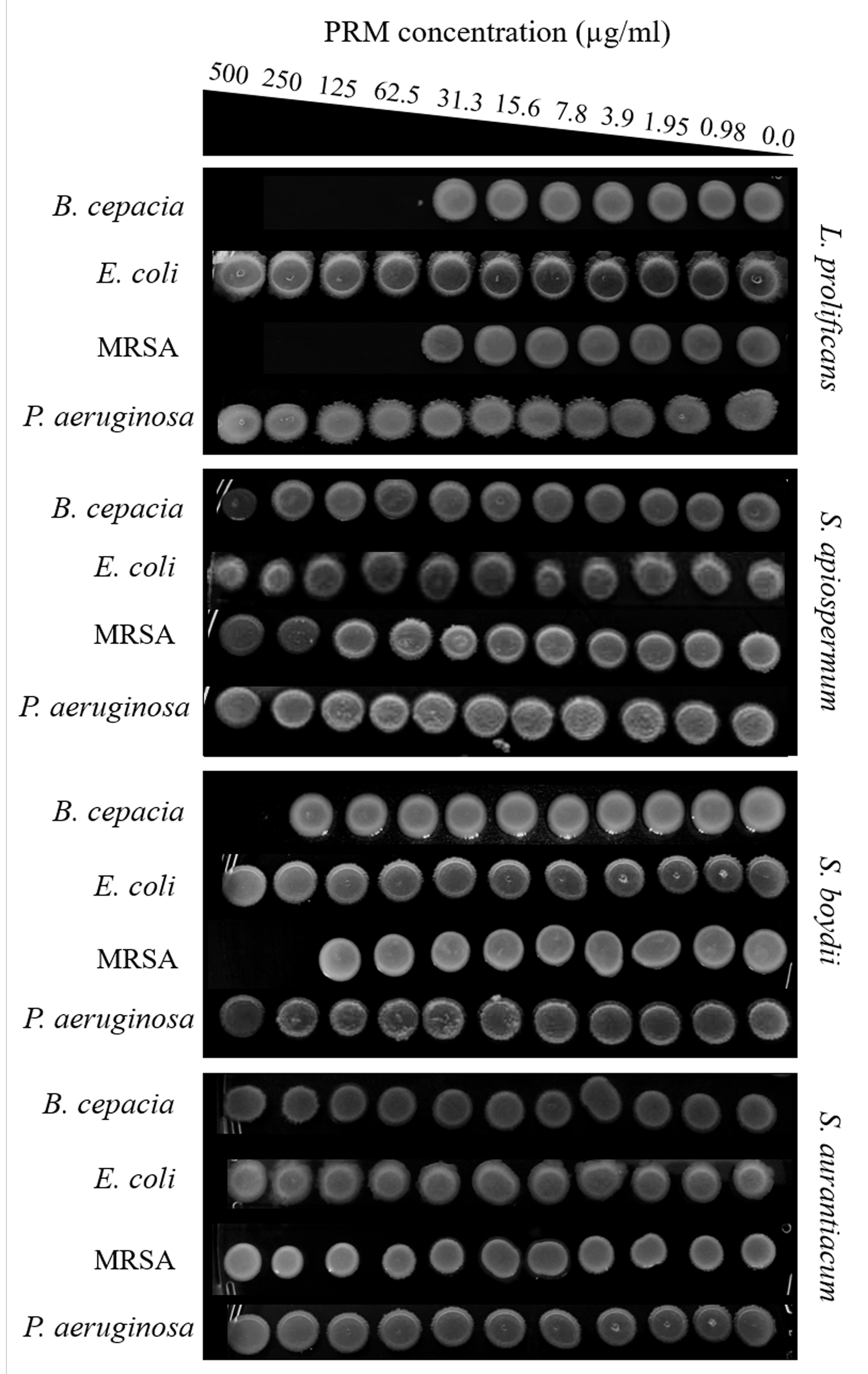

Supplement: Supplementary Material 1 — Growth of B. cepacia, E. coli, MRSA and P. aeruginosa in the presence of different concentrations (0.98 – 500 μg/ml) of PRM isolated from L. prolificans, S. apiospermum, S. boydii and S. aurantiacum. [file Image_1.tif]
